# Selective Extraction Process and Characterization of Antioxidant Phenolic Compounds from *Pereskia aculeata* Leaves Using UPLC-ESI-Q-TOF-MS/MS

**DOI:** 10.1021/acsomega.4c05652

**Published:** 2024-08-21

**Authors:** Simone Silva Jacobsen, Fernanda Caroline Knob, Anna Paulla Simon, Tatiane Luiza Cadorin Oldoni

**Affiliations:** Department of Chemistry, Federal Technological University of Paraná (UTFPR), Pato Branco, PR 85503-390, Brazil

## Abstract

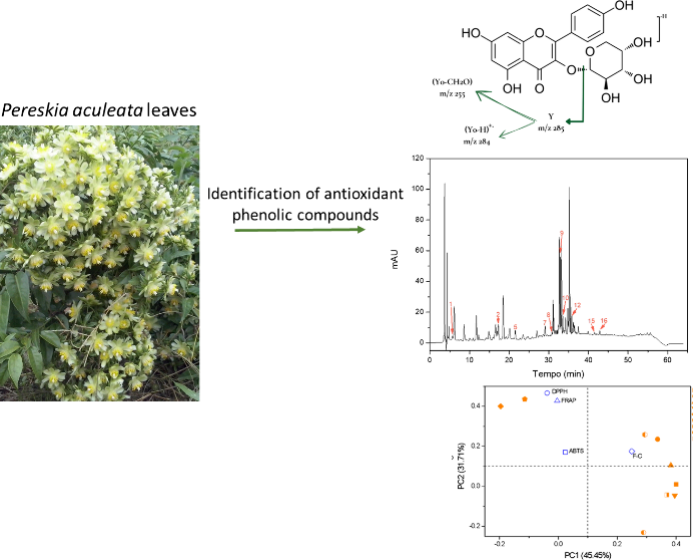

This study innovates in comparing biological activities and chemical composition obtained from extracts and fractions from *Pereskia aculeate* leaves. Seven extracts and five fractions were produced by conventional successive solid–liquid extraction coupled with simultaneous bioguided purification using solvents of distinct polarities. A comparative analysis was conducted between these purified fractions and the original extracts to elucidate potential improvements in the bioactivity. The extract and fractions were evaluated using the ABTS, DPPH, FRAP, and Folin–Ciocalteau methods and HPLC-DAD and UHPLC-ESI-Q-TOF-MS/MS evaluated chemical composition. The fractions obtained from the hydroalcoholic extract showed better results, with the acetone fraction (Fr-Ace) exhibiting enhanced bioactivity, especially in the FRAP (1095 μmol of FeSO_4_/g) antioxidant capacity method. The results demonstrated that medium to high polarity solvents were the most effective in extracting bioactive phenolic compounds, with rutin being the predominant compound. The sequential hydroalcoholic fractionation (SHF) method extracted a greater variety of compounds, including vanillic acid and cinnamic acid, which were reported for the first time in *P. aculeate* leaves. The identified compounds by UPLC-Q-TOF-MS/MS included flavonoids derived from quercetin, isorhamnetin, and kaempferol, phenolic acids, and their derivatives. Quercetin-3-*O*-xyloside, kaempferol-3-*O*-arabinoside, trehalose, feruloyltyramine, malyngic acid, pinellic acid, and 16-hydroxy-9-oxooctadeca-10,12,14-trienoic acid were identified for the first time in *P. aculeata* leaves.

## 1 Introduction

Plants have been utilized for medicinal purposes for over 4000 years. Among the notable active metabolites isolated from plants are morphine, codeine, atropine, and caffeine.^1^ The type and quantity of these metabolites vary depending on the species and are influenced by biotic and abiotic factors.^2^ An important class of secondary metabolites is the phenolic compound. They perform different functions in the plant, like structural support protection against biotic and abiotic stress and pigmentation, among others.^3^

The medicinal potential of phenolic compounds has attracted attention in recent years. For example, recent research has demonstrated the virucidal properties of B-type procyanidin condensed tannins derived from (−)-epicatechin against SARS-CoV-2. This compound was isolated from the methanol leaf extract of Kratom (*Mitragyna speciosa*) demonstrating the significant therapeutic potential of these compounds.^4^

However, given the critical roles of these metabolites play, analyzing bioactive substances is often complex and time-consuming process, primarily due to the low quantities in which plants produce them. Thus, bioguided assays offer a more efficient approach by isolating and characterizing fractions with biological activity, thereby streamlining the identification of new compounds and determining their chemical structures.^5,6^

*Pereskia aculeata* Miller, commonly known as ora-pro-nóbis, which means “pray for us” in Latin, is native to tropical America and is notable for being a hardy and easily propagated plant, widely distributed throughout Brazil.^7^ Its leaves are consumed for their high protein content, classifying it as a Non-Conventional Edible Plant (NCEP). Additionally, it is widely used in traditional medicine for treating burns and promoting ulcer healing, among other conditions.^8^

Studies have shown that *P. aculeata* extracts exhibit promising results for acute and chronic anti-inflammatory dermatitis and antioxidant activities.^9^ Some extracts have demonstrated the potential to inhibit microbial growth of Gram-positive and Gram-negative bacteria.^10^ Most of these activities can be attributed to the chemical composition of *P. aculeata*, which predominantly includes phenolic compounds, tannins, alkaloids, and flavonoids. Compounds such as quercetin, caffeic acid derivatives, kaempferol, isorhamnetin glycoside derivatives, caftaric acid, lutein, β-carotene, and α-carotene have already been identified in *P. aculeata* leaves.^7,11^

Despite evidence of the antioxidant activity in *P. aculeata* leaves, there is a lack of studies dedicated to optimizing the extraction of compounds from this plant to achieve a profile of its bioactive components. A comprehensive profile is essential for maximizing its potential as a food or functional/medicinal plant source. Therefore, this study aims to identify the most effective extraction method for extracting antioxidant compounds from *P. aculeata*, concentrating these bioactive compounds using a bioassay-guided purification method, determine the chemical composition using UPLC-ESI-Q-TOF-MS/MS, and optimize the chromatographic method to identify and quantify 17 phenolic compounds accurately.

## 2 Results and Discussion

This study aims to determine whether a hydroalcoholic extraction (HE) followed by fractionation process produces fractions with superior antioxidant potential compared to direct solvent extraction. Both methods are based on conventional solid–liquid extraction, where the efficacy of the solvent is influenced mainly by the solubility of the target analytes and their interactions with other components in the sample.

### 2.1 Antioxidant Activity of Hydroalcoholic Extract, Fractions, and Extracts

Plant extracts are complex matrices that require different tests to determine antioxidant activity accurately. These assays operate under different experimental conditions, such as single electron transfer (SET) and hydrogen atom transfer (HAT).^12^ The fractions and extracts obtained from *P. aculeata* were evaluated using the Folin–Ciocalteu (F–C) and Ferric Reducing Antioxidant Power (FRAP) assays, which are based on the SET mechanism, as well as the DPPH and ABTS assays, which incorporate both SET and HAT mechanisms. For both extraction methods, sequential hydroalcoholic fractionation (SHF) and direct solvent extraction (DSE), a significant variation was observed with respect to the solvent used (Table 1).

**Table 1:** Antioxidant Activity of HE, Fractions, and Extracts, Obtained from *P. aculeata* Leaves, Evaluated by the F–C, FRAP, DPPH, and ABTS Assays^a^

Sample	F–C assay (mg GAE/g)	FRAP assay (μmol FeSO_4_/g)	DPPH assay (μmol TEAC/g)	ABTS assay (μmol TEAC/g)
Extraction Method: SHF				
HE (EtOH:H_2_O)	75.1 ± 1.7 aA	865 ± 70 bB	239 ± 20 aA	882 ± 53 aA
Fr-Hex	41.3 ± 3.3 eB	273 ± 20 eB	50.3 ± 4.6 eA	484 ± 33 dA
Fr-DCM	46.8 ± 3.4 dA	536 ± 41 dB	39.5 ± 2.1 fA	610 ± 15 cA
Fr-EtOAc	68.4 ± 3.0 bA	939 ± 35 bA	99.9 ± 4.3 dA	517 ± 37 dA
Fr-Ace	54.5 ± 2.9 cA	1095 ± 77 aA	155 ± 8.0 bA	577 ± 32 cA
Fr-But	57.9 ± 3.5 cA	696 ± 69 cA	130 ± 20 cA	831 ± 24 bA
Extraction method: DSE				
E-Hex	44.5 ± 2.2 bA	341 ± 9.7 fA	5.2 ± 0.44 eB	44.1 ± 6.6 eB
E-DCM	47.1 ± 3.8 bA	606 ± 32 eA	4.9 ± 0.22 eB	76.1 ± 3.3 dB
E-EtOAc	70.4 ± 0.15 aA	706 ± 34 dB	106 ± 2.2 cA	249 ± 19 bB
E-Ace	33.2 ± 2.3 cB	902 ± 51 cB	104 ± 8.2 c,dB	242 ± 16 bB
E-But	32.1 ± 0.15 cB	633 ± 14 eB	94.2 ± 6.9 dB	199 ± 8.3 cB
E-EtOH	49.7 ± 2.8 b	1439 ± 37 a	242 ± 16 a	334 ± 26 a
E-EtOH:H_2_O	46.7 ± 2.8 bB	1203 ± 26 bA	164 ± 8.5 bB	374 ± 14 aB

a Values are expressed as mean ± SD (*n* = 9). Mean values with different lowercase letters, in the same column, for the same method of extraction, are significantly different by the Tukey’s test (*p* ≤ 0.05). Mean values with different uppercase letters, for samples obtained with the same solvent, are significantrly different by the Student’s *t* test (*p* < 0.05).

The results for the hydroalcoholic extract (HE) and fractions produced by the sequential hydroalcoholic fractionation (SHF) method demonstrated a higher capacity to reduce the Folin–Ciocalteu reagent, indicating a higher phenolic compound content. The highest content observed was 75.1 mg of GAE/g for the HE, followed by a fraction obtained from ethyl acetate (70.4 mg of GAE/g). In contrast, extracts obtained by the direct solvent extraction (DSE) method showed phenolic contents ranging from 32.1 to 70.4 mg of GAE/g for E-But and E-EtOAc, respectively (Table 1). A significant difference in phenolic compound content was observed upon comparison of the SHF and DSE methods.

Torres et al.^13^ reported total phenolic content (TPC) values ranging from 3.71 to 60.09 mg of GAE/g, with the highest values found in pressurized liquid extraction (PLE) using ethanol and water. Additionally, the phenolic content values were lower when Soxhlet extraction was used with hexane and ethanol solvents (4.6 and 15 mg of GAE/g). Furthermore, the TPC results obtained in this study are higher than those reported by Cruz et al.^14^ for *P. aculeata* leaves, which ranged between 26 and 66 mg of GAE/g.

From the evaluated assays, the FRAP method was the only antioxidant assay that showed superior results for the direct solvent extraction, yielding 1439 μmol of FeSO_4_/g for extract produced using ethanol. In both extraction methods, hexane was the least effective solvent for extracting compounds with iron reduction capacity. In the FRAP assay, the fractions obtained from HE (SHF method) with the highest antioxidant capacities were Fr-Ace (1095 μmol of FeSO_4_/g) and Fr-EtOAc (939 μmol of FeSO_4_/g). For the DSE extraction method, E-EtOH was able to extract 4.2 times more bioactive compounds than E-Hex (341 μmol of FeSO_4_/g) (Table 1).

Solvents such as ethanol and water, which are more polar, have a greater capacity to extract phenolic compounds that contain hydroxyl (OH) groups, providing polarity to the molecule. In the case of the DSE method, the higher antioxidant capacity observed using the Folin–Ciocalteau assay with ethyl acetate could be attributed to its efficiency in extracting specific subclasses of phenolic compounds.

According to results obtained from HPLC-DAD (Table 3), the phenolic compounds were identified in higher amounts: caffeic acid, coumaric acid, vanillic acid, ferulic acid, quercetin, and kaempferol. Meanwhile, the highest antioxidant capacity observed using the FRAP assay with ethyl alcohol (DSE) and acetone (SHF) as solvents indicates that these solvents were more effective in extracting flavonoids derived from quercetin such as isoquercetin and rutin (Table 3).

Our findings were superior to those of Torres et al.,^13^ who reported FRAP values ranging from 0.09 to 0.17 mmol of TE/g when employing hexane solvent in the Soxhlet method and ethanol solvent in the Pressurized Liquid Extraction (PLE) method, respectively. Our results obtained using the ethanol by the DSE method yielded 87% higher results (1.2 mmol of FeSO_4_/g).

For both radical scavenging methods, the HE and fractions obtained from the SHF method showed higher and significant differences than those obtained from the DSE method. For the DPPH assay, the hydroalcoholic extract (HE) showed a higher antioxidant capacity (239 μmol of TEAC/g), while fractions ranged from 39 to 155 μmol of TEAC/g. The results from extracts of the DSE extraction method varied from 5.19 μmol of TEAC/g (E-Hex) to 242 μmol of TEAC/g (E-EtOH) (Table 1).

The highest antioxidant capacities for the ABTS assay were observed in the HE (882 μmol of TEAC/g) and Fr-But (831 μmol of TEAC/g). For the DSE extraction method, the extracts E-EtOH:H_2_O (374 μmol of TEAC/g) and E-EtOH (334 μmol of TEAC/g) exhibited the highest antioxidant activities, with no significant variation. Both extraction methods revealed that the lowest antioxidant capacities were associated with less polar solvents, such as hexane and dichloromethane, yielding 76.1 μmol of TEAC/g for Fr-DCM and 44.1 μmol of TEAC/g for E-Hex (Table 1).

Despite their similar aims, the significant differences in the absolute antioxidant capacity values measured by the DPPH-TEAC and ABTS-TEAC assays are due to intrinsic factors. The DPPH assay primarily measures hydrogen-donating antioxidants at 517 nm, while the ABTS assay measures hydrogen- and electron-donating antioxidants at 734 nm.^12^

The DPPH assay uses organic solvents like methanol or ethanol, affecting antioxidant solubility and reactivity differently than the aqueous environment typically used in the ABTS assay. Additionally, the kinetics of the reactions also play a role. The phenolic compounds with multiple hydroxyl groups react more readily with the ABTS radical cation than with the DPPH radical, leading to higher values in the ABTS assay.^15^

Overall, the fractions and extracts obtained from solvents with medium to high polarities, such as ethanol, ethyl acetate, and acetone, were more efficient in extracting bioactive phenolic compounds. Therefore, more polar compounds, such as flavonoids, which possess −OH groups in their chemical structure, enhance their affinity for polar solvents. According to Torres et al.,^13^ although limited studies have assessed the antioxidant activity of water-based extracts from *Pereskia* sp. leaves, the water-based extracts demonstrated superior antioxidant activities across the evaluated methods.

### 2.2 Profile of Phenolic Compounds of HE, Fractions, and Extracts Using HPLC-DAD

High-performance liquid chromatography (HPLC) was optimized for the separation of 17 phenolic compounds, of which 9 belong to the group of phenolic acids (gallic acid, chlorogenic acid, vanillic acid, caffeic acid, *p*-coumaric acid, ferulic acid, isochlorogenic acid, salicylic acid, and cinnamic acid) and 8 flavonoids (catechin, epicatechin, rutin, isoquercitrin, astragalin, myricetin, quercetin, and kaempferol).

The quality parameters were evaluated to assess the efficacy of the method optimization employed to separate the 17 analyzed compounds. The capacity factor (*k*) values were calculated based on the time the mixture’s constituents passed through the column and should ideally range between 1 and 20 for complex mixtures such as the analyzed sample. The obtained values ranged from 0.85 to 13.3 (Table 2). While the *k* value for gallic acid is slightly below this range at 0.85, the other compounds exhibit suitable *k* values ranging from 4.7 (catechin) to 13.3 (kaempferol). These values indicate that the compounds interacted with the stationary phase for an adequate duration, allowing effective separation, which indicates a good interaction of the analytes with the stationary phase over an appropriate interval.

**Table 2:** Optimized Parameters Obtained for the Calibration Curve at 30 mg/L^a^

Compound	RT (min)	*N*	*H* (mm)	*k*	α		Rs	
Gallic acid	6.0	13104	0.019	0.85	5.5		46.8	
Catechin	18.4	52957	0.0047	4.7		1.1		3.6
Chlorogenic acid	19.5	59780	0.0042	5.0	1.1		5.7	
Vanillic acid	21.4	60207	0.0042	5.6		1.0		1.8
Caffeic acid	22.0	63734	0.0039	5.8	1.2		11.5	
Epicatechin	25.8	118703	0.0021	7.0		1.2		12.7
Coumaric acid	29.5	177722	0.0014	8.1	1.1		9.8	
Ferulic acid	32.1	264113	0.00095	8.9		1.0		1.5
Rutin	32.4	466417	0.00054	9.0	1.0		4.2	
Isoquercetin	33.2	442225	0.00057	9.3		1.0		4.9
Isochlorogenic acid A	34.2	518703	0.00048	9.6	1.0		6.1	
Astragalin	35.3	554162	0.00045	9.9		1.0		2.8
Salicylic acid	36.0	203401	0.0012	10.1	1.0		3.3	
Myricetin	37.0	324900	0.00077	10.4		1.1		19.5
Quercetin	41.7	575667	0.00043	11.9	1.0		4.5	
Cinnamic acid	42.8	433571	0.00058	12.2		1.1		12.9
Kaempferol	46.2	468667	0.00053	13.3				

a RT: retention time, *N*: number of theoretical plate number, *H*: theoretical plate height, *k*: retention factor, α: selectivity, Rs: resolution.

Furthermore, selectivity (α) and resolution (Rs) were evaluated to quantify the system’s capacity to differentiate and separate its components. Ideally, selectivity (α) should be greater than 1.2, indicating the system’s ability to differentiate between components effectively. Although some values, such as the α value of 1.0 between rutin and isoquercitrin, fell short of this ideal, they remained close to the target.

Resolution (Rs), which measures the separation between two bands with adequate baseline width, should ideally be ≥1.5. In our method, Rs values ranged from 1.5 to 46.8, indicating effective separation of all components and demonstrating the method’s efficiency.

Additionally, the efficiency of the employed chromatographic method was evaluated by assessing the number of theoretical plates (*N*) and the height equivalent to the theoretical plate (*H*). These parameters relate to the method’s efficiency, particularly in separating a complex mixture composed of 17 compounds, many with similar chemical properties.

**Figure 1:**
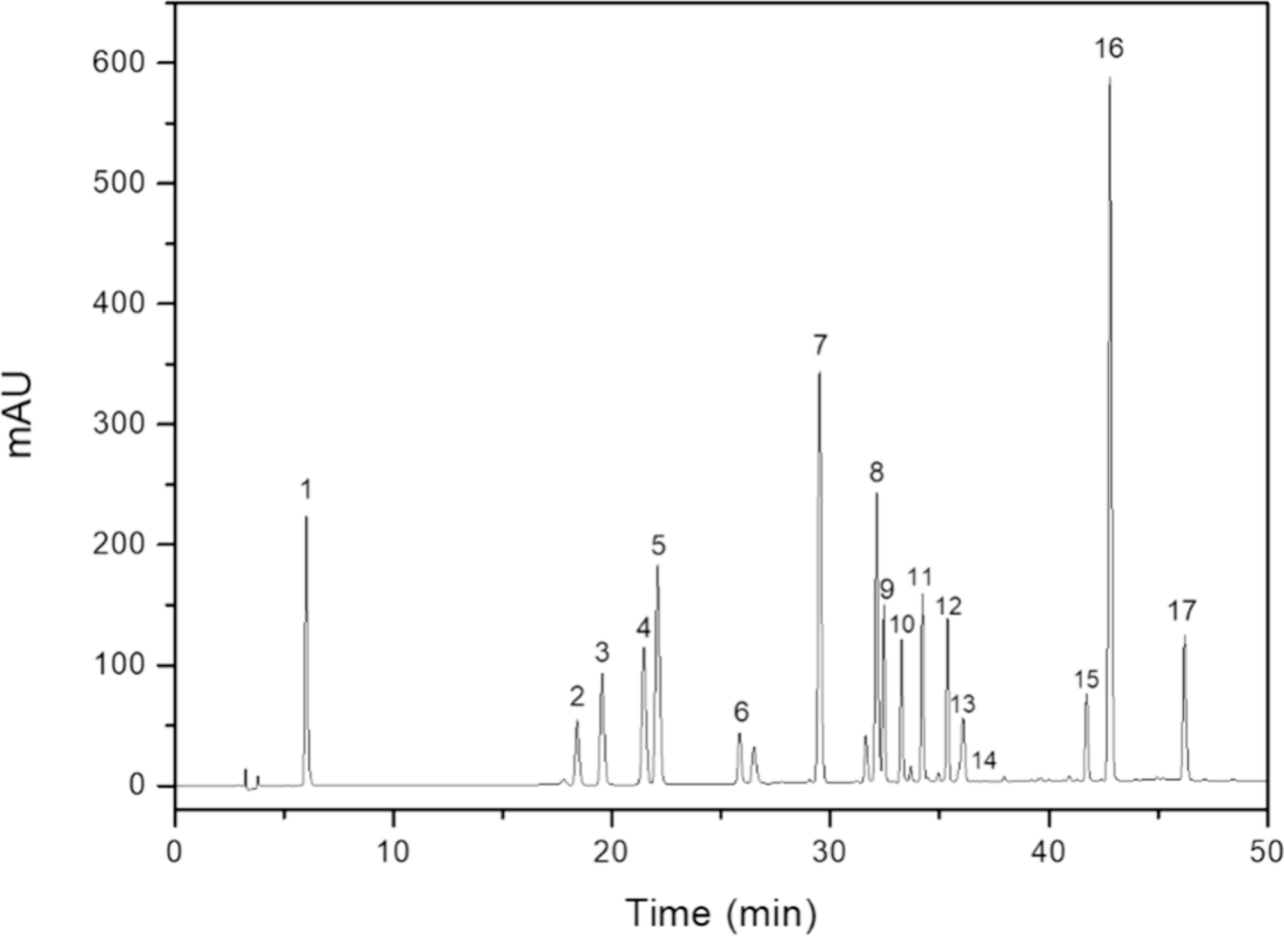
Chromatogram obtained for standard mix at 280 nm. (1) gallic acid; (2) catechin; (3) chlorogenic acid; (4) vanillic acid; (5) caffeic acid; (6) epicatechin; (7) coumaric acid; (8) ferulic acid; (9) rutin; (10) isoquercetin; (11) isochlorogenic acid A; (12) astragalin; (13) salicylic acid; (14) myricetin; (15) quercetin; (16) cinnamic acid; (17) kaempferol.

The less polar compounds, such as cinnamic acid and kaempferol, exhibit longer retention times than the more polar compounds, such as gallic acid and catechin. This behavior is related to the interaction of these compounds with both the mobile and stationary phases. Less polar compounds interact more strongly with the reversed-phase column, whereas more polar compounds interact more strongly with the polar mobile phase. The chromatogram of the phenolic compounds used to construct the calibration curve is presented in Figure 1.

**Figure 2:**
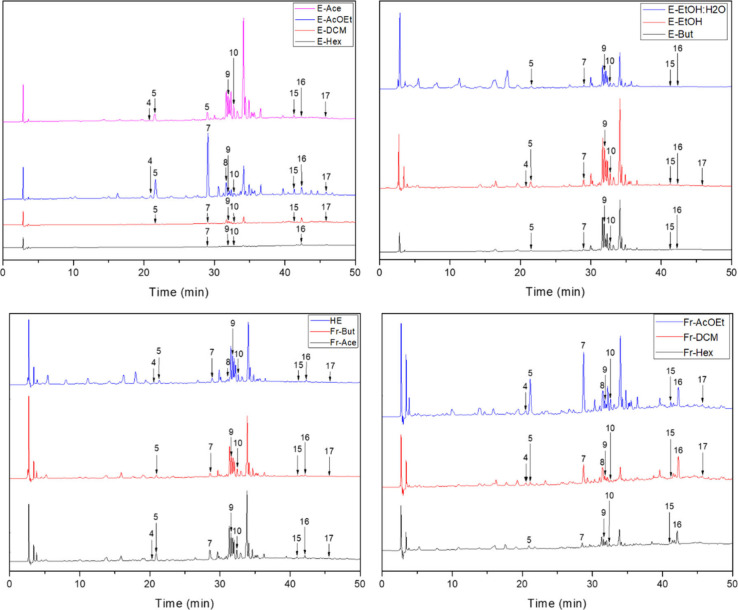
Chromatograms for the hydroalcoholic extract (EH), Fractions, and Extracts, obtained at 280 nm. (4) Vanillic acid; (5) caffeic acid; (7) coumaric acid; (8) ferulic acid; (9) rutin; (10) isoquercetin; (15) quercetin; (16) cinnamic acid; (17) kaempferol.

The extraction efficiency was evaluated by detecting and quantifying the bioactive substances in the HE, fractions, and extracts of *P. aculeata* (Figures 1 and 2). Vanillic acid, caffeic acid, coumaric acid, ferulic acid, rutin, isoquercetin, quercetin, cinnamic acid, and kaempferol were identified in the extracts and fractions of the leaves. Among these, the flavonoid rutin was the predominant compound in almost all of the analyzed samples (Table 3).

**Table 3:** Content of Phenolic Compounds Identified on HE, Fractions, and Extracts, Obtained from *P. aculeata* Leaves^a^

Sample	Vanillic acid	Caffeic acid	Coumaric acid	Ferulic acid	Rutin	Isoquercetin	Quercetin	Cinnamic acid	Kaempferol
	(mg of compound g^–1^ of extract or fraction)								
Extraction method: SHF									
HE	<LQ	0.4 ± 0.01 cA	0.4 ± 0.01 cA	<LQ	6.6 ± 0.13 aA	1.6 ± 0.03 abA	0.1 ± 0.01 bA	<LQ	<LQ
Fr-Hex	<LD	0.1 ± 0.01 eA	0.1 ± 0.01 dA	<LD	0.9 ± 0.04 cA	0.2 ± 0.03 d	0.04 ± 0.01 cA	0.2 ± 0.01 c	<LD
Fr-DCM	0.2 ± 0.01 b	0.1 ± 0.01 eA	0.5 ± 0.03 cA	0.5 ± 0.02 b	0.5 ± 0.01 cA	<LQ	0.04 ± 0.01 cA	0.3 ± 0.02 aA	0.5 ± 0.15 aA
Fr-EtOAc	0.3 ± 0.04 aB	1.5 ± 0.02 aA	1.6 ± 0.03 aB	0.6 ± 0.01 aB	0.9 ± 0.04 cA	1.2 ± 0.03 cA	0.3 ± 0.02 aB	0.3 ± 0.01 bA	0.4 ± 0.02 aA
Fr-Ace	0.2 ± 0.02 b	0.8 ± 0.07 bA	0.7 ± 0.08 bA	<LD	5.3 ± 0.53 bA	1.9 ± 0.19 aB	0.3 ± 0.05 aA	0.1 ± 0.01 d	0.1 ± 0.02 bA
Fr-But	<LD	0.3 ± 0.02 dA	0.2 ± 0.03 dA	<LD	4.9 ± 0.28 bB	1.5 ± 0.13 bcB	0.09 ± 0.02 bcA	<LQ	0.06 ± 0.02 bA
Extraction method: DSE									
E-Hex	<LD	<LD	<LQ	<LD	0.2 ± 0.01 eB	<LQ	<LD	<LQ	<LD
E-DCM	<LD	0.04 ± 0.01 fB	0.08 ± 0.01 eB	<LD	0.6 ± 0.10 dA	<LQ	0.04 ± 0.01 cA	0.2 ± 0.01 bB	0.06 ± 0.01 bB
E-EtOAc	0.6 ± 0.02 aA	1.9 ± 0.01 aA	3.4 ± 0.02 aA	0.7 ± 0.01 aA	0.6 ± 0.04 dB	0.5 ± 0.03 eB	0.6 ± 0.02 aA	0.2 ± 0 aB	0.4 ± 0.02 aA
E-Ace	<LQ	0.6 ± 0.03 cA	0.4 ± 0.03 cB	<LD	4.5 ± 0.04 cB	2.2 ± 0.06 bA	0.3 ± 0.02 bA	<LQ	0.06 ± 0.02 bA
E-But	<LD	0.1 ± 0.01 eB	0.1 ± 0.01 dA	<LD	7.8 ± 0.07 bA	1.9 ± 0.03 cA	0.04 ± 0.02 cA	<LQ	<LD
E-EtOH	<LQ	0.8 ± 0.03 b	0.6 ± 0.02 b	<LD	11.1 ± 0.07 a	3.0 ± 0.11 a	0.3 ± 0.05 b	<LQ	0.10 ± 0.01 b
E-EtOH:H_2_O	<LD	0.3 ± 0.01 dB	0.1 ± 0.01 dB	<LD	4.4 ± 0.06 cB	1.0 ± 0.01 dB	0.1 ± 0.01 cA	<LQ	<LD

a Values are expressed as mean ± SD (*n* = 3). Mean values with different lowercase letters, in the same column, for the same method of extraction, are significantly different by the Tukey’s test (*p* ≤ 0.05). Mean values with different uppercase letters, for samples obtained with the same solvent, are significantly different by the Student’s *t* test (*p* < 0.05). LD = Limit of detection. LQ = Limit of quantification.

From the results obtained by HPLC-DAD, among the 17 evaluated standards, 9 phenolic compounds, including 4 flavonoids and 5 phenolic acids, were identified in the leaves of *P. aculeata*. Quercetin, rutin, caffeic acid, and coumaric acid were present in all extracts except those produced with hexane solvent (Fr-Hex and E-Hex). Notably, rutin was the most abundant compound across all samples. The direct solvent extraction (DSE) method yielded the E-EtOH extract with the highest rutin content (11 mg/g), followed by the E-But extract (7.8 mg/g). In the sequential hydroalcoholic fractionation (SHF) method, the hydroalcoholic extract (HE) exhibited the highest rutin concentration at 6.6 mg/g, followed by Fr-Ace and Fr-But at 5.3 and 4.9 mg/g, respectively. Isoquercetin was not detected in extracts produced with hexane and dichloromethane solvents, which were the least polar extracts.

When solvent extraction properties were evaluated, it is possible to conclude that ethanol, acetone, and butanol extracted glycosylated quercetin derivatives more efficiently with their strong hydrogen bonding capabilities and varying polarities. Ethanol and acetone, exhibiting high polarity and strong hydrogen bonding, effectively dissolved these compounds. Butanol, though less polar, facilitated extraction due to its intermediate polarity and hydrogen bonding.^16^

Cruz et al.,^14^ in their optimization of extraction conditions, quantified the phenolic compounds rutin, quercetin, and caffeic acid in concentrations ranging from 0.44 to 1.8 mg/g, 0.025 to 0.072 mg/g, and 0.24 to 0.50 mg/g, respectively, with the highest concentrations observed in hydroalcoholic extracts containing 60% water and 40% ethanol. Similarly, Garcia et al.^11^ quantified rutin at a concentration of 3.5 mg/g and a caffeic derivative at 0.5 mg/g and identified ten phenolic compounds with caftaric acid as the major compound. The content of all phenolic compounds identified in this study was higher than those reported by the mentioned studies.

Despite the complexity of the hydroalcoholic extract (HE), it was possible to achieve a good separation with the identification and quantification of several phenolic compounds. The SHF method extracted a greater variety of compounds than the DSE method. Vanillic acid, ferulic acid, cinnamic acid, and kaempferol were not quantified in HE; however, these compounds were detected in fractions obtained from HE fractionation. For instance, vanillic acid and cinnamic acid were reported for the first time in *P. aculeata* leaves, with higher amounts found in E-EtOAc and Fr-DCM, respectively. Coumaric acid was predominant in E-EtOAc, which exhibited the greatest variety of compounds, whereas E-Hex showed the least variety. Other studies have also reported the presence of p-coumaric acid, ferulic acid, kaempferol, and isoquercetin in *P. aculeata* leaves.^11,13,17^

The ethyl acetate, with its moderate polarity and balanced hydrophilic–lipophilic profile, acts as a hydrogen bond acceptor, enhancing its ability to dissolve the polar hydroxyl groups and the less polar aromatic rings of these phenolic acids. This makes ethyl acetate particularly effective for extracting compounds with mixed polarities.

Dichloromethane (DCM), a nonpolar solvent with low hydrogen bonding capacity, is effective at dissolving hydrophobic and slightly polar compounds. Its nonpolar nature is advantageous for extracting the hydrophobic components of cinnamic acid and vanillic acid, particularly their aromatic rings. Although DCM does not interact through hydrogen bonding, its ability to solubilize nonpolar components complements the extraction process.

These findings underscore the importance of bioguided fractionation in obtaining fractions rich in bioactive compounds with potential antioxidant properties. Using different solvents in the fractionation process proved to be an effective strategy for enhancing the concentrations of phenolic compounds. This bioguided purification process demonstrates efficiency and potential for developing antioxidant-rich extracts from *P. aculeata* leaves.

It is well-known that genetic effects as well as environmental and physiological conditions significantly influence the composition of bioactive compounds in plants, impacting the complexity of cactus composition. These factors need further investigation, particularly in the most consumed species of the Pereskioideae subfamily.^17^

**Figure 3:**
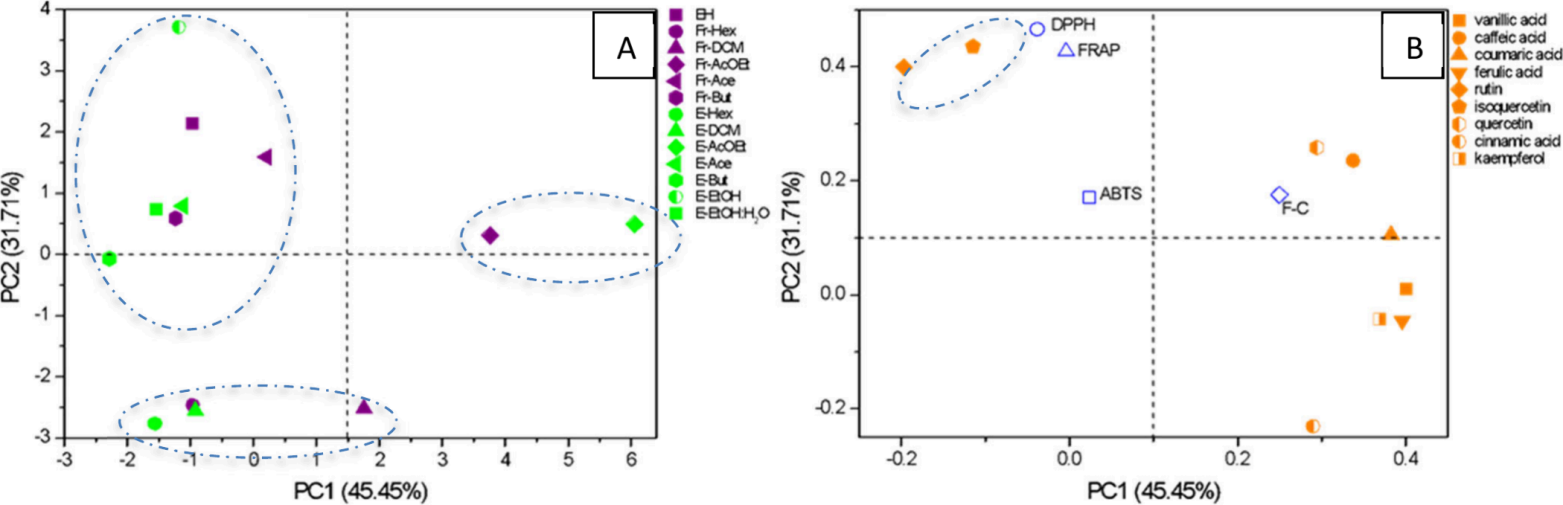
Scores (A) and loading (B) plot of the HE, fractions, and extracts of the *P. aculeata* leaves.

To highlight the similarities and differences among solvents, extracts, and fractions, the results obtained from HPLC quantification and antioxidant activity tests were analyzed using principal component analysis (PCA), a multivariate analysis tool. The two principal components (PCs) together accounted for 77.16% of the total data variance (PC1: 45.45% and PC2: 31.71%), making them suitable for visualizing the correlations between the samples (Figure 3).

The samples were grouped according to the solvent used, indicating the extraction capabilities of each solvent. The PCA revealed a separation into three major groups, as shown in Figure 3A. The first group consisted of samples obtained with medium and high polarity solvents, a second group with samples obtained using ethyl acetate, and a third group with samples obtained with low polarity solvents. Solvents with high and medium polarities clustered distinctly from hexane and dichloromethane, with separation predominantly observed along PC2. This suggests that these solvents extract different classes of compounds with varying antioxidant activities.

The loading plot (Figure 3B) indicates that rutin and isoquercetin are key in distinguishing samples obtained with more polar solvents from the others. Additionally, these samples exhibited the highest ABTS, DPPH, and FRAP activities. Rutin is known for its extensive pharmacological uses such as antimicrobial, antifungal, and antiallergic properties, especially as a free radical scavenger.^11^ Furthermore, rutin is a potent α-glucosidase inhibitor, which may be beneficial for the treatment of type 2 diabetes, as evidenced by the findings of Astiti et al.^18^

Rutin and isoquercetin have multiple hydroxyl groups, enhancing their polarity. These hydroxyl groups form hydrogen bonds with polar solvents, increasing the solubility and extraction efficiency. Nonpolar solvents cannot form these interactions, resulting in poor extraction. Thus, polar solvents align well with the polar nature of rutin and isoquercetin, facilitating more efficient extraction through hydrogen bonding and dipole–dipole interactions.^19^

The fraction (Fr-EtOAc) and extract (E-EtOAc) were separated from the other samples in the scores plot. Analysis of the loading plot showed that these two samples contained the highest levels of vanillic acid, caffeic acid, coumaric acid, ferulic acid, quercetin, and kaempferol. In terms of antioxidant activity, these samples demonstrated the highest values in the Folin–Ciocalteu (F–C) assay. Still, samples obtained with hexane and dichloromethane presented the lowest content of most compounds, except for Fr-DCM, which showed the highest amount of cinnamic acid, as observed in the loadings plot.

The findings of this study suggest that the choice of solvent and extraction method directly influences the final composition of an extract and, consequently, its bioactivity. In all of the evaluated antioxidant assays, extracts and fractions obtained with more polar solvents exhibited the best bioactivities. Comparing the chemical profiles of these extracts and fractions revealed that they were all characterized by high rutin and isoquercetin contents.

Based on the results obtained for antioxidant activity, the HE and the fractions obtained from its purification exhibited the highest antioxidant potential, as measured by antioxidant assays. Notably, the DPPH and ABTS radical scavenging methods and the total phenolic content assay highlighted the superior antioxidant activities. Additionally, the levels of phenolic compounds such as *p*-coumaric acid, rutin, cinnamic acid, and kaempferol were also higher in HE and its fractions compared to those of the crude extracts. Consequently, HE and its fractions were subjected to chemical composition analysis using the UHPLC-ESI-QTOF-MS/MS technique, which provided detailed insights into their phenolic profiles and further supported the observed antioxidant activities.

### 2.3 UPLC-ESI-QTOF-MS/MS

The identified compounds were numbered according to their order of elution. They consisted predominantly of flavonoids derived from quercetin, isorhamnetin, and kaempferol and their glycosylated flavonoids, phenolic acids, and their derivatives (Table 4). Quercetin-3-*O*-xyloside, kaempferol-3-*O*-arabinoside, trehalose, feruloyltyramine, malyngic acid, pinellic acid, and 16-hydroxy-9-oxooctadeca-10,12,14-trienoic acid were identified for the first time in *P. aculeata* leaves.

**Table 4:** UPLC-ESI-QTOF-MS/MS Data of the Compounds Identified on HE and Fractions, Obtained from *P. aculeata* Leaves^a^

N°	Compound	RT	Molecular formula	*m*/*z* Experimental	*m*/*z* Theoretical	Fragments	Sample
1	Sucrose	2.7	C_12_H_22_O_11_	341.1122	341.1090	179.0601 (100)	Fr-Hex
						161.0491 (40.82)	Fr-DCM
						143.0382 (39.58)	
2	Trehalose	2.8	C_12_H_22_O_11_	341.1105	341.1090	89.0257 (47.17)	HE
						179.0561 (28.30)	Fr-Ace
							Fr-But
3	Citric acid	4.1	C_6_H_8_O_7_	191.0209	191.0197	111.0098 (100)	HE
						87.0082 (43.21)	Fr-Hex
							Fr-DCM
4	Caftaric acid	20.7	C_13_H_12_O_9_	311.0436	311.0403	179.0358 (100)	HE
						149.0153 (53.97)	
						135.0454 (49.21)	
5	Caffeic acid	23.5	C_9_H_8_O_4_	179.0390	179.0360	135.0488 (100)	Fr-EtOAc
6	Quercetin-Rhamnoside-Hexoside-Pentoside	29.6	C_32_H_38_O_20_	741.1913	741.1920	300.0277 (100)	HE
							Fr-Hex
7	Coumaric acid	30.1	C_9_H_8_O_3_	163.0439	163.0422	119.0534 (100)	Fr-EtOAc
8	Rutin	30.7	C_27_H_30_O_16_	609.1493	609.1460	271.0273 (93.02)	HE
						255.0333 (48.84)	Fr-Hex
						300.0339 (36.96)	Fr-DCM
							Fr-Ace
							Fr-But
9	Isoquercetin	31.9	C_21_H_20_O_12_	463.0904	463.0880	300.0305 (100)	HE
						271.0267 (75.61)	Fr-Hex
						301.0370 (50)	Fr-EtOAc
							Fr-Ace
							Fr-But
10	Ferulic acid	31.9	C_10_H_10_O_4_	193.0542	193.0510	134.0396 (100)	Fr-DCM
						178.0328 (31.82)	Fr-EtOAc
11	Isorhamnetin-3-*O*-galactoside-6″-rhamnoside	33.1	C_28_H_32_O_16_	623.1640	623.1620	271.0221 (71.05)	HE
						299.0247 (49.09)	Fr-Hex
							Fr-DCM
							Fr-EtOAc
							Fr-Ace
							Fr-But
12	Quercetin-3-*O*-xyloside	33.4	C_20_H_18_O_11_	433.0800	433.0780	300.0303 (100)	HE
						301.0327 (82.14)	Fr-Hex
						271.0220 (68.63)	Fr-EtOAc
							Fr-Ace
							Fr-But
13	Isorhamnetin-3-*O*-glucoside	34.3	C_22_H_22_O_12_	477.1058	477.1038	315.0461 (100)	HE
						314.0458 (66.67)	Fr-Hex
						271.0271 (36.14)	Fr-EtOAc
							Fr-Ace
							Fr-But
14	Kaempferol-3-*O*-arabinoside	35.1	C_20_H_18_O_10_	417.0907	417.0830	284.0382 (76.47)	Fr-EtOAc
						255.0368 (50)	
						227.0382 (44)	
15	Azelaic acid	35.8	C_9_H_16_O_4_	187.0998	187.0976	125.1010 (100)	Fr-Hex
							Fr-DCM
							Fr-EtOAc
							Fr-Ace
							Fr-But
16	Feruloyltyramine	39.0	C_18_H_19_NO_4_	312.1286	312.1241	148.0547 (100)	Fr-DCM
						178.0576 (72.50)	Fr-EtOAc
17	Quercetin	40.6	C_15_H_10_O_7_	301.0419	301.0350	151.0071 (100)	Fr-EtOAc
						179.0041 (44.86)	
18	Malyngic acid	44.2	C_18_H_32_O_5_	327.2215	327.2180	211.1361 (50)	Fr-Hex
						229.1475 (3673)	Fr-DCM
							Fr-EtOAc
19	Pinellic acid	47.4	C_18_H_34_O_5_	329.2384	329.2312	211.1359 (29.63)	Fr-DCM
						229.1515 (15.58)	Fr-EtOAc
20	16-Hydroxy-9-oxooctadeca-10,12,14-trienoic acid	48.1	C_18_H_28_O_4_	307.1964	307.1910	185.1222 (100)	Fr-DCM
						235.1345 (34.62)	Fr-EtOAc
						121.0656 (55.17)	Fr-Ace

a The *m/z* values were obtained in negative mode (M–H)^−^.

Compounds **6** (quercetin-ramnoside-hexoside-pentoside), **8** (rutin), **9** (isoquercetin), and **12** (quercetin-3-*O*-xyloside) were identified as quercetin derivatives. All of these compounds showed a characteristic precursor ion resulting from the elimination of the glycosylated portion [Y]^−^ and the H radical, yielding the radical ion [Y – H] at *m*/*z* 300.^20^

Compounds **8** and **9** were identified in all samples analyzed, except for the ethyl acetate and dichloromethane fraction, respectively. These compounds produced precursor ion at *m*/*z* 271 due to the loss of [Y – CHO].^20^ Rutin and isoquercetin have already been identified in *P. aculeata* leaves.^11,21^

Compound **6** was identified in the hydroalcoholic extract and in the hexane fraction of *P. aculeate* as previously described by Cruz et al.^14^ Compound **12** was identified in all fractions analyzed, except in the dichloromethane fraction. Quercetin and its glycosylated derivatives, such as quercetin-*O*-pentoside-*O*-rutinoside and quercetin-*O*-pentoside-*O*-hexoside, were previously reported in samples of *P. aculeata* leaves.^22^

Compound **14** (kaempferol-3-*O*-arabinoside) was identified only in the ethyl acetate fraction at 35.1 min with precursor ion *m*/*z* 417.0907 and the characteristic fragment ion resulting from the elimination of the glycosylated portion [Y] and the H radical, producing the radical ion [Y – H] at *m*/*z* 284. Fragment ions were observed at *m*/*z* 255, related to the loss of the COH group [Y – CH_2_O].^20^ Compound **11** (isorhamnetin-3-*O*-galactoside-6″-ramnoside) was observed in all fractions analyzed, while compound **13** (isorhamnetin-3-*O*-glucoside) was observed in all fractions, except in the dichloromethane fraction, and both compounds were identified as isorhamnetin derivatives.

Compound **13** shows a precursor ion at *m*/*z* 477.1078 and a fragment ion at *m*/*z* 315 [Y], indicative of the loss of the glycoside moiety. Subsequently, a fragment ion at *m*/*z* 271 was detected, corresponding to the sequential loss of a CH_3_ radical, followed by the loss of a CHO group.^23^ Compound **11** shows a fragment ion at *m*/*z* 299, corresponding to the loss of the two sugar moieties and a CH_3_ group.

Some organic acids were identified in the analyzed samples. Compound **4** was identified at 20.7 min, in HE, and presented a precursor ion at *m*/*z* 311, which produced the fragment ions at *m*/*z* 179, 149, and 135 characteristic of the fragmentation of caftaric acid. This compound has already been reported in other works as a major compound in *P. aculeata* leaves.^11,14^ Caftaric acid has been investigated for its antioxidant, anti-inflammatory, and antimutagenic potentials.

Compounds **5** (caffeic acid), **7** (coumaric acid), and **10** (ferulic acid) were identified at 23.5, 30.1, and 31.9 min, respectively, in the ethyl acetate fraction. Compounds **5** and **7** present fragment ions corresponding to carbon dioxide loss at *m*/*z* 135 and 119 [M – H – CO_2_], respectively. Compound **10** shows two fragment ions at *m*/*z* values of 178 and 134 corresponding to loss of the methyl group [M – H – CH_3_] and loss of the methyl group and carbon dioxide [M – H – CO_2_ – CH_3_], respectively.

The caffeic acid found in the leaves of *P. aculeata* has the potential to inhibit the generation of reactive oxygen species, again emphasizing the use of this plant as an alternative source of phenolic compounds. Caffeic, coumaric, and ferulic acids also have antioxidant, antimicrobial, and anti-inflammatory activities, helping to prevent several chronic noncommunicable diseases, such as cancer and diabetes.^24^

Compound **15** (azelaic acid) was identified in all samples analyzed except in the HE. This compound was also identified by Souza et al.^22^ in a study with *P. aculeata* leaves collected in different seasons. It is suggested that the fragment ion at *m*/*z* 125 corresponds to the loss of one molecule of water and carbon dioxide [M – H – CO_2_ – H_2_O].

Compound **2** (trehalose) was identified in the hydroalcoholic extract and the butanol and acetone fractions with precursor ion at *m*/*z* 341, which fragment at *m*/*z* 179 corresponding to the C_6_H_11_O_6_ group. Trehalose is used in various products, including pharmaceuticals, food, and cosmetics. This sugar has been the focus of numerous studies because it has very peculiar physical and chemical properties, which differentiate it from other sugars.^25^

Compound **16** (feruloyltyramine) was identified at 39 min in the dichloromethane and ethyl acetate fractions, respectively. An ion at *m*/*z* 148 characteristic of the [M – H – C_9_H_9_O_2_] fragment is observed. Trans-feruloyltyramine and 7′-ethoxy-trans-feruloyltyramine have been identified in other species of the Cactaceae family, as well as other nitrogen-containing compounds (phenethylamines, isoquinoline alkaloids, and betalains)^17^

Compounds **18**, **19**, and **20** were tentatively identified as malyngic acid, pinellic acid, and 16-hydroxy-9-oxooctadeca-10,12,14-trienoic acid. The three compounds are octadecatrienoic acid, which is characterized as polyunsaturated long-chain fatty acids. The compounds were identified in less polar fractions. Malyngic acid and pinellic acid have been previously described by Mirzaei et al.^26^ in *Potentilla reptans* L. root extracts but have not yet been reported in the leaves of *P. aculeata*.

## 3 Conclusions

The hydroalcoholic extract (HE) and sequential hydroalcoholic fractionation (SHF) method had the highest phenolic content and antioxidant capacities, superior to that shown by the direct solvent extraction (DSE) method. Medium to high polarity solvents were most effective in extracting bioactive phenolic compounds with rutin being the predominant compound. The SHF method extracted a greater variety of compounds, including vanillic acid and cinnamic acid, which were reported for the first time in *Pereskia aculeata* leaves.

The identified compounds included flavonoids derived from quercetin, isorhamnetin, and kaempferol as well as phenolic acids, and their derivatives. Quercetin-3-*O*-xyloside, kaempferol-3-*O*-arabinoside, trehalose, feruloyltyramine, malyngic acid, pinellic acid, and 16-hydroxy-9-oxooctadeca-10,12,14-trienoic acid were identified for the first time in *P. aculeata* leaves.

## 4 Methods

### 4.1 Chemicals

DPPH (2,2-diphenyl-1-picrylhydrazyl hydrate), Trolox (6- hydroxy-2,5,7,8-tetramethylchroman-2-carboxylic acid), 2,4,6-tri(2-pyridyl)-s-triazine (TPTZ), gallic acid, catechin, chlorogenic acid, vanillic acid, caffeic acid, epicatechin, transcoumaric acid, ferulic acid, rutin, isoquercetin, isochlorogenic acid A, astragaline, salicylic acid, myricetin, quercetin, cinnamic acid, and kaempferol were obtained from Sigma-Aldrich (St. Louis, MO, USA). Solvents of HPLC grade were acquired from J. T. Baker (Phillipsburg, NJ, USA); ethanol, Na_2_CO_3_, Folin–Ciocalteu reagent, and phosphoric acid were purchased from Dinâmica (Diadema, SP, Brazil).

### 4.2 Plant Material and Extraction Process

#### 4.2.1 Plant Material, Extraction with Ethanol Solution, and Fractionation

In August 2021, the leaves of *Pereskia aculeata* were provided by the company Proteios Nutrição Funcional Ltd. from Ribeirão Branco-São Paulo/Brazil. The leaves were received dry and milled. Plant material (500 g) was extracted with 3 L of ethanol:water (80:20, v/v) solution, under stirring at 99.5 rpm for 24 h, at room temperature, and then filtered. This procedure was repeated for 5 days, with the replacement of the extractor solvent and the combination of the filtrates. After that, the solvent ethanol was evaporated using a rotary evaporator IKA RV 10 (Staufen, Alemanha) under reduced pressure at 40 °C, and the water was removed using lyophilization (Liobras L101, São Carlos, Brasil). This process yielded a concentrated solid of 40 g, which was designated as the hydroalcoholic extract (HE) and used in the subsequent steps.

The HE (40 g) was submitted to solid–liquid extraction and conducted exhaustively using 320 mL of each solvent: hexane (Fr-Hex, 3.3 g), dichloromethane (Fr-DCM, 2.0 g), ethyl acetate (Fr-EtOAc, 0.2 g), acetone (Fr-Ace, 0.36 g), and butanol (Fr-But, 0.4 g). Each solvent was replaced at intervals of 24 h, filtered, combined, and concentrated in a rotary evaporator. This process was named sequential hydroalcoholic fractionation (SHF)

#### 4.2.2 Direct Solvent Extraction (DSE)

Plant material (50 g) was subjected to solid–liquid extraction and conducted exhaustively using 200 mL of solvents: hexane (E-Hex, 2.0 g), dichloromethane (E-DCM, 0.7 g), ethyl acetate (E-EtOAc, 0.2 g), acetone (E-Ace, 0.3 g), butanol (E-But, 0.1 g), ethanol (E-EtOH, 1.18 g), and a mixture of ethanol and water (80:20, v/v) (E-EtOH:H_2_O, 2.6 g). Each solvent was replaced at 24 h intervals at room temperature. The extracts were then filtered and concentrated by using a rotary evaporator. The E-EtOH:H_2_O extract was further lyophilized to remove the water.

### 4.3 Antioxidant Activity

#### 4.3.1 Reducing Capacity of the Folin–Ciocalteu Reagent (F–C Assay)

The F–C assay assessed the reducing capacity of the sample.^27^ The reaction mixture included 0.5 mL of the sample, 2.5 mL of diluted F–C reagent (1:10), and 2.0 mL of 4% Na_2_CO_3_. The test tubes were kept at room temperature in the dark for 2 h. Absorbance was measured at 740 nm using a PerkinElmer Lambda 25 spectrophotometer (PerkinElmer Inc., Waltham, MA, USA). The calibration curve was created by plotting the absorbance against gallic acid concentration ranging from 5 to 100 mg/L, and the results were expressed as gallic acid equivalents (mg of GAE/g).

#### 4.3.2 Ferric Reducing Antioxidant Power (FRAP)

The FRAP reagent was prepared by mixing 25 mL of acetate buffer (300 mmol/L, pH 3.6), 2.5 mL of 2,4,6-tripyridyl-s-triazine (TPTZ) solution (10 mmol/L in 40 mmol/L HCl), and 2.5 mL of FeCl_3_ solution (20 mmol/L in water). The reaction mixture was comprised of 100 μL of sample and 3.0 mL of FRAP reagent.^28^ The test tubes were incubated at 37 °C in a water bath for 30 min. Absorbance was measured at 593 nm using an aqueous FeSO_4_ solution (200 to 2000 μmol/L) as a standard.

#### 4.3.3 Scavenging of Radical DPPH (2,2-Diphenyl-1-picrylhydrazyl) Assay

The reaction mixture included 0.5 mL of the sample, 3.0 mL of ethanol:water (80:20 v/v), and 0.3 mL of 0.5 mmol/L DPPH in ethanol solution. The test tubes were kept at room temperature in the dark for 1 h.^29^ Absorbance was measured at 517 nm, using Trolox for calibration (15 to 100 μmol/L), and the results were expressed as μmol of Trolox equivalent antioxidant capacity (TEAC/g).

#### 4.3.4 Scavenging of Radical ABTS (2,2′-Azinobis(3-ethylbenzothiazoline-6-sulfonic acid)) Assay

The ABTS radical was generated by reacting 7 mmol/L ABTS stock solution with 140 mmol/L potassium persulfate, and the mixture was kept in the dark at room temperature for 16 h.^30^ This ABTS^•+^ solution was then diluted with ethanol to absorb 0.700 ± 0.050 at 734 nm. To conduct the assay, 3.0 mL of the ABTS^•+^ solution was mixed with 30 μL of the sample. The test tubes were incubated in the dark at room temperature for 6 min. The absorbance was subsequently measured at 734 nm. Trolox was used as the standard for calibration (100 to 2000 μmol/L), and the results were reported as μmol of Trolox equivalent antioxidant capacity (TEAC/g).

#### 4.3.5 High Performance Liquid Chromatography with Diode Array Detector (HPLC-DAD)

The chromatographic analysis was conducted using a Varian 920-LC high-performance liquid chromatograph (Varian Inc., Palo Alto, CA, EUA) with a ZORBAX Eclipse Plus C18 column (250 mm × 4.6 mm, 5 μm), maintained at a constant temperature of 30 °C. The mobile phase consisted of a mixture of water and acetic acid (98:2 v/v) (solvent A) and acetonitrile, water, and acetic acid (40:58:2 v/v) (solvent B). The flow rate was set at 1 mL/min in gradient mode, starting with 5% solvent B, increasing to 25% B in 20 min, 85% B in 40 min, maintaining 85% B at 45 min, increasing to 95% B at 48 min, holding 95% B at 51 min, reducing to 5% B at 54 min, and maintaining with 5% B at 64 min. The injection volume of the sample was 10 μL. The extracts and fractions were characterized by comparing retention times (RT) and characteristic absorption at λ 280/360 nm. The standards used included gallic acid, catechin, chlorogenic acid, vanillic acid, caffeic acid, epicatechin, coumaric acid, ferulic acid, rutin, isoquercetin, isochlorogenic acid A, astragalin, salicylic acid, myricetin, quercetin, trans-cinnamic acid, and kaempferol. Peak areas were measured with concentrations ranging from 1 to 50 mg/L for quantification.^6^

### 4.4 UPLC-ESI-Q-TOF-MS/MS

The MS/MS experiments used a Q-TOF Maxis 3G (Bruker Daltonics, MA, EUA) high-resolution mass spectrometer with a Q-TOF geometry and an electrospray ionization source. The ionization source operated in negative ionization mode, set to 4500 V with an end plate offset potential of −500 V. Drying gas parameters were set to 8 L/min at 180 °C with a nebulizer gas pressure of 4 bar. Data acquisition spanned an *m*/*z* range of 50 to 1800 at a rate of 5 Hz, with the 5 most intense ions automatically selected for fragmentation (Auto MS/MS) using a stepping program with collision energy ranging from 15 to 40 eV.^31^ The conditions for chromatographic separation were the same as those optimized in section 4.3.5.

### 4.5 Statistical Analysis

Statistical analyses were conducted using Jamovi software version 2.3.28. Comparisons between data sets were made using a one-way analysis of variance (ANOVA) with a 95% confidence level. Tukey’s posthoc test was applied to groups showing statistically significant differences, with significance defined as *p* < 0.05. Additionally, the antioxidant activity variables and HPLC quantification data were analyzed using principal component analysis (PCA). PCA was performed with PLS_toolbox 3.0 in MATLAB 7.0.1 (MathWorks). Before PCA, data were preprocessed using autoscaling. Graphs were created using Origin 8.5 software.

## Supporting Information Available

Analytical data obtained by high-performance liquid chromatography (HPLC) for the phenolic compounds analyzed in the study. This data provides detailed information on the retention times, maximum wavelength of absorption, calibration curve, limit of detection, and limit of quantification, ensuring the accuracy and reproducibility of the results. (PDF)

## References

[ref1] Dutra R. C.; Campos M. M.; Santos A. R. S.; Calixto J. B. Medicinal Plants in Brazil: Pharmacological Studies, Drug Discovery. Challenges and Perspectives. Pharmacol Res. 2016, 112, 4–29. 10.1016/j.phrs.2016.01.021. 26812486

[ref2] Tungmunnithum D.; Thongboonyou A.; Pholboon A.; Yangsabai A. Flavonoids and Other Phenolic Compounds from Medicinal Plants for Pharmaceutical and Medical Aspects: An Overview. Medicines 2018, 5 (3), 93 10.3390/medicines5030093. 30149600 PMC6165118

[ref3] Albuquerque B. R.; Heleno S. A. 8.; Oliveira M. B. P. P.; Barros L.; Ferreira I. C. F. R. Phenolic Compounds: Current Industrial Applications, Limitations and Future Challenges. Food and Function; Royal Society of Chemistry: 2021; pp 14–29. 10.1039/d0fo02324h. 33242057

[ref4] Sureram S.; Chutiwitoonchai N.; Pooprasert T.; Sangsopha W.; Limjiasahapong S.; Jariyasopit N.; Sirivatanauksorn Y.; Khoomrung S.; Mahidol C.; Ruchirawat S.; Kittakoop P. Discovery of Procyanidin Condensed Tannins of (−)-Epicatechin from Kratom, Mitragyna Speciosa, as Virucidal Agents against SARS-CoV-2. Int. J. Biol. Macromol. 2024, 273, 133059 10.1016/j.ijbiomac.2024.133059. 38866269

[ref5] Hochheim S.; Pacassa Borges P.; Boeder A. M.; Scharf D. R.; Simionatto E. L.; Yamanaka C. N.; Alberton M. D.; Guedes A.; de Cordova C. M. M. A Bioguided Approach for the Screening of Antibacterial Compounds Isolated From the Hydroalcoholic Extract of the Native Brazilian Bee’s Propolis Using Mollicutes as a Model. Front Microbiol 2020, 11, 558 10.3389/fmicb.2020.00558. 32318040 PMC7154171

[ref6] Oldoni T. L. C.; Merlin N.; Karling M.; Carpes S. T.; Alencar S. M.; Morales R. G. F.; Silva E. A.; Pilau E. J. Bioguided Extraction of Phenolic Compounds and UHPLC-ESI-Q-TOF-MS/MS Characterization of Extracts of Moringa Oleifera Leaves Collected in Brazil. Food Research International 2019, 125, 108647 10.1016/j.foodres.2019.108647. 31554035

[ref7] Hoff R.; Daguer H.; Deolindo C. T. P.; de Melo A. P. Z.; Durigon J. Phenolic Compounds Profile and Main Nutrients Parameters of Two Underestimated Non-Conventional Edible Plants: Pereskia Aculeata Mill. (Ora-pro-Nóbis) and Vitex Megapotamica (Spreng.) Moldenke (Tarumã) Fruits. Food Research International 2022, 162, 112042 10.1016/j.foodres.2022.112042. 36461259

[ref8] Bolson M.; Hefler S. R.; Dall’Oglio Chaves E. I.; Gasparotto Junior A.; Cardozo Junior E. L. Ethno-Medicinal Study of Plants Used for Treatment of Human Ailments, with Residents of the Surrounding Region of Forest Fragments of Paraná. Brazil. J. Ethnopharmacol 2015, 161, 1–10. 10.1016/j.jep.2014.11.045. 25482361

[ref9] Pinto N. D. C. C.; Machado D. C.; Da Silva J. M.; Conegundes J. L. M.; Gualberto A. C. M.; Gameiro J.; Moreira Chedier L.; Castañon M. C. M. N.; Scio E. Pereskia Aculeata Miller Leaves Present in Vivo Topical Anti-Inflammatory Activity in Models of Acute and Chronic Dermatitis. J. Ethnopharmacol 2015, 173, 330–337. 10.1016/j.jep.2015.07.032. 26226436

[ref10] Milião G. L.; de Oliveira A. P. H.; Soares L. de S.; Arruda T. R.; Vieira É. N. R.; Leite Junior B. R. de C. Unconventional Food Plants: Nutritional Aspects and Perspectives for Industrial Applications. Future Foods 2022, 5, 100124 10.1016/j.fufo.2022.100124.

[ref11] Garcia J. A. A.; Corrêa R. C. G.; Barros L.; Pereira C.; Abreu R. M. V.; Alves M. J.; Calhelha R. C.; Bracht A.; Peralta R. M.; Ferreira I. C. F. R. Phytochemical Profile and Biological Activities of “Ora-pro-Nobis” Leaves (Pereskia Aculeata Miller), an Underexploited Superfood from the Brazilian Atlantic Forest. Food Chem. 2019, 294, 302–308. 10.1016/j.foodchem.2019.05.074. 31126467

[ref12] Huang D.; Boxin O. U.; Prior R. L. The Chemistry behind Antioxidant Capacity Assays. J. Agric. Food Chem. 2005, 53 (6), 1841–1856. 10.1021/jf030723c. 15769103

[ref13] Torres T. M. S.; Álvarez-Rivera G.; Mazzutti S.; Sánchez-Martínez J. D.; Cifuentes A.; Ibáñez E.; Ferreira S. R. S. Neuroprotective Potential of Extracts from Leaves of Ora-pro-Nobis (Pereskia Aculeata) Recovered by Clean Compressed Fluids. J. Supercrit. Fluids 2022, 179, 105390 10.1016/j.supflu.2021.105390.

[ref14] Cruz T. M.; Santos J. S.; do Carmo M. A. V.; Hellström J.; Pihlava J. M.; Azevedo L.; Granato D.; Marques M. B. Extraction Optimization of Bioactive Compounds from Ora-pro-Nobis (Pereskia Aculeata Miller) Leaves and Their in Vitro Antioxidant and Antihemolytic Activities. Food Chem. 2021, 361, 130078 10.1016/j.foodchem.2021.130078. 34023692

[ref15] Prior R. L.; Wu X.; Schaich K. Standardized Methods for the Determination of Antioxidant Capacity and Phenolics in Foods and Dietary Supplements. J. Agric. Food Chem. 2005, 53 (10), 4290–4302. 10.1021/jf0502698. 15884874

[ref16] El Mannoubi I. Impact of Different Solvents on Extraction Yield, Phenolic Composition, in Vitro Antioxidant and Antibacterial Activities of Deseeded Opuntia Stricta Fruit. Journal of Umm Al-Qura University for Applied Sciences 2023, 9 (2), 176–184. 10.1007/s43994-023-00031-y.

[ref17] da Silveira Agostini-Costa T. Bioactive Compounds and Health Benefits of Pereskioideae and Cactoideae: A Review. Food Chem. 2020, 327, 126961 10.1016/j.foodchem.2020.126961. 32422230

[ref18] Astiti M. A.; Jittmittraphap A.; Leaungwutiwong P.; Chutiwitoonchai N.; Pripdeevech P.; Mahidol C.; Ruchirawat S.; Kittakoop P. LC-QTOF-MS/MS Based Molecular Networking Approach for the Isolation of α-Glucosidase Inhibitors and Virucidal Agents from Coccinia Grandis (L.) Voigt. Foods 2021, 10 (12), 3041 10.3390/foods10123041. 34945591 PMC8701318

[ref19] Sasidharan S.; Chen Y.; Saravanan D.; Sundram K. M.; Latha L. Y. Extraction, Isolation And Characterization Of Bioactive Compounds From Plants’ Extracts. Afr J. Complement Altern Med. 2010, 8 (1), 1–10. 10.4314/ajtcam.v8i1.60483. PMC3218439 22238476

[ref20] Kumar S.; Singh A.; Kumar B. Identification and Characterization of Phenolics and Terpenoids from Ethanolic Extracts of Phyllanthus Species by HPLC-ESI-QTOF-MS/MS. J. Pharm. Anal 2017, 7 (4), 214–222. 10.1016/j.jpha.2017.01.005. 29404041 PMC5790687

[ref21] Macedo M. C. C.; Silva V. D. M.; Serafim M. S. M.; da Veiga Correia V. T.; Pereira D. T. V.; Amante P. R.; da Silva A. S. J.; de Oliveira Prata Mendonça H.; Augusti R.; de Paula A. C. C. F. F.; Melo J. O. F.; Pires C. V.; Fante C. A. Elaboration and Characterization of Pereskia Aculeate Miller Extracts Obtained from Multiple Ultrasound-Assisted Extraction Conditions.. Metabolites 2023, 13 (6), 691 10.3390/metabo13060691. 37367849 PMC10300959

[ref22] Souza L. F.; Caputo L.; De Barros I. B. I.; Fratianni F.; Nazzaro F.; De Feo V. Pereskia aculeata Muller (Cactaceae) Leaves: Chemical Composition and Biological Activities. Int. J. Mol. Sci. 2016, 17 (9), 1478 10.3390/ijms17091478. 27598154 PMC5037756

[ref23] Sriseadka T.; Wongpornchai S.; Rayanakorn M. Quantification of Flavonoids in Black Rice by Liquid Chromatography- Negative Electrospray Ionization Tandem Mass Spectrometry. J. Agric. Food Chem. 2012, 60 (47), 11723–11732. 10.1021/jf303204s. 23121250

[ref24] Gomes de Souza P.; Azeredo D. R. P.; da Silva T. T. C.; Carneiro C. da S.; Junger Teodoro A.; Menezes Ayres E. M. Food Neophobia, Risk Perception and Attitudes Associations of Brazilian Consumers towards Non-Conventional Edible Plants and Research on Sale Promotional Strategies. Food Research International 2023, 167, 112628 10.1016/j.foodres.2023.112628. 37087204

[ref25] Millinia B. L.; Mashithah D.; Nawatila R.; Kartini K. Microencapsulation of Roselle (Hibiscus Sabdariffa L.) Anthocyanins: Effects of Maltodextrin and Trehalose Matrix on Selected Physicochemical Properties and Antioxidant Activities of Spray-Dried Powder. Future Foods 2024, 9, 100300 10.1016/j.fufo.2024.100300.

[ref26] Mirzaei H.; Jekő J.; Cziáky Z.; Jahanshahi M.; Zengin G.; Enayati A. LC–MS/MS Phytochemical Profiling, Antioxidant Activity, and Enzyme Inhibitory of Potentilla Reptans L. Root: Computational Studies and Experimental Validation. Process Biochemistry 2024, 137, 30–40. 10.1016/j.procbio.2023.12.013.

[ref27] Singleton V. L.; Orthofer R.; Lamuela-Raventós R. M. Analysis of Total Phenols and Other Oxidation Substrates and Antioxidants by Means of Folin-Ciocalteu Reagent. Methods Enzymol 1999, 299, 152–178. 10.1016/S0076-6879(99)99017-1.

[ref28] Benzie I. F. F.; Strain J. J. The Ferric Reducing Ability of Plasma (FRAP) as a Measure of ‘“ Antioxidant Power ”’: The FRAP Assay. Anal. Biochem. 1996, 239, 70–76. 10.1006/abio.1996.0292. 8660627

[ref29] Brand-Williams W.; Cuvelier M. E.; Berset C. Use of a Free Radical Method to Evaluate Antioxidant Activity. LWT - Food Science and Technology 1995, 28 (1), 25–30. 10.1016/S0023-6438(95)80008-5.

[ref30] Re R.; Pellegrini N.; Proteggente A.; Pannala A.; Yang M.; Rice-Evans C. Antioxidant Activity Applying an Improved ABTS Radical Cation Decolorization Assay. Free Radic Biol. Med. 1999, 26 (9/10), 1231–1237. 10.1016/S0891-5849(98)00315-3. 10381194

[ref31] Prasniewski A.; da Silva C.; Ayres B. R. B.; Silva E. A. da; Pilau E. J.; Nani B. D.; Rosalen P. L.; Oldoni T. L. C. Characterization of Phenolic Compounds by UHPLC-QTOF-MS/MS and Functional Properties of Syzygium Malaccense Leaves. South African Journal of Botany 2021, 139, 418–426. 10.1016/j.sajb.2021.01.036.

