# Supplementary material for: Selective Extraction Process and Characterization of Antioxidant Phenolic Compounds from Pereskia aculeata Leaves Using UPLC-ESI-Q-TOF-MS/MS
Source: ACS Omega. 2024 Aug 21;9(35):37374–85. doi: 10.1021/acsomega.4c05652 (PMC11375806; doi:10.1021/acsomega.4c05652)
Supplement: Supplementary file 1 — ao4c05652_si_001.pdf [file ao4c05652_si_001.pdf]

Selective extraction process and characterization of antioxidant phenolic compounds from *Pereskia aculeata* leaves using UPLC-ESI-Q-TOF-MS/MS

Simone S. Jacobsen[a], Fernanda C. Knob[a], Anna P. Simon[a], Tatiane L. C. Oldoni[a*]

Supplementary material

Table S1 - Analytical data obtained by HPLC for phenolic compounds

**Nº**	**Compound**	**RT (min)**	**$\lambda_{máx}$ (nm)**	**Calibration curve**	**R^2**	**LD (mg/L)**	**LQ (mg/L)**
1	**Gallic acid**	6.0	271	y = 0.4062x - 0.0525	0.999	0.12	0.39
2	**Catechin**	18.4	278	y = 0.0887x + 0.0123	0.999	0.41	1.38
3	**Chlorogenic acid**	19.6	324	y = 0.2214x - 0.0524	0.999	0.23	0.75
4	**Vanillic acid**	21.4	260	y = 0.5431x - 0.2364	0.999	0.46	1.54
5	**Caffeic acid**	22.1	322	y = 0.523x + 0.094	0.998	0.08	0.3
6	**Epicatechin**	25.8	278	y = 0.0938x + 0.0132	0.999	1.13	3.75
7	**Coumaric acid**	29.5	309	y = 0.6808x - 0.1171	0.999	0.10	0.32
8	**Ferulic acid**	32.1	322	y = 0.5847x - 0.0002	0.999	0.06	0.19
9	**Rutin**	32.4	254/354	y = 0.1116x - 0.0148	0.998	0.08	0.27
10	**Isoquercetin**	33.2	253/354	y = 0.1556x - 0.0194	0.999	0.53	1.77
11	**Isochlorogenic acid A**	34.2	324	y = 0.395x + 1.2583	0.996	0.03	0.11
12	**Astragalin**	35.3	263/345	y = 0.3791x + 0.9288	0.999	0.31	1.04
13	**Salicylic acid**	36.0	301	y = 0.5522x - 0.3646	0.999	0.30	0.50
14	**Myricetin**	37.0	371	y = 0.2156x - 0.0828	0.998	1.00	5.00
15	**Quercetin**	41.7	252/371	y = 0.2068x + 0.0207	0.998	0.08	0.3
16	**Cinnamic acid**	42.8	276	y = 1.2068x - 0.2695	0.999	0.33	1.08
17	**Kaempferol**	46.2	262/366	y = 0.2477x - 0.0582	0.999	0.10	0.33

RT: retention time. Λmáx: wavelength max. LD: limit of detection (mg/L). LQ: limit of quantification (mg/L).
